# Reducing Psychosocial Risk Factors and Improving Employee Well-Being in Emergency Departments: A Realist Evaluation

**DOI:** 10.3389/fpsyg.2021.728390

**Published:** 2022-02-03

**Authors:** Anne Nathal de Wijn, Margot Petra van der Doef

**Affiliations:** Institute of Psychology, Health, Medical, and Neuropsychology Unit, Leiden University, Leiden, Netherlands

**Keywords:** stress management intervention, process variables, process evaluation, Psychosocial Safety Climate (PSC), employee participation, burnout – professional, nurses, emergency department

## Abstract

This study reports the findings of a 2.5 year intervention project to reduce psychosocial risks and increase employee well-being in 15 emergency departments in the Netherlands. The project uses the psychosocial risk management approach “PRIMA” which includes cycles of risk assessment, designing and implementing changes, evaluating changes and adapting the approach if necessary. In addition, principles of participative action research were used to empower the departments in designing and implementing their own actions during the project. Next to determining overall effects, the study aims to assess potential moderators including the level of intervening (organization-directed or multilevel), process variables (the number and fit of actions to risk factors, communication and employee participation) and partaking in a Psychosocial Safety Climate intervention offered during the second half of the project. The results of linear mixed-model analyses showed that all job factors improved with the exception of autonomy, which did increase halfway the project but not when considering the entire timeframe. In addition, work engagement decreased and symptoms of burnout remained stable. Emergency departments that implemented more fitting actions, communicated better and involved their employees more in the process, had more favorable changes in job factors and more stable well-being. More activity (based on the number of actions implemented) and a multilevel approach regarding stress management did not lead to greater improvements. The Psychosocial Safety Climate intervention was effective in improving Psychosocial Safety Climate, but a longer follow-up period seems required to evaluate its effect on job factors and well-being. Overall, the project resulted in positive changes in most job factors, and its findings emphasize the importance of process variables in stress management interventions. Longer follow-up and higher quality multilevel interventions (including professional support for employees with stress-related complaints) seem essential to also improve well-being.

## Introduction

High levels of work-related stress have been related to mental and physical problems (Colligan and Higgins, 2006), reduced productivity (Letvak and Buck, 2008), more absenteeism (Schmidt et al., 2019) and higher turnover intentions (Mosadeghrad et al., 2011; Nei et al., 2015). According to a review on studies performed in Western Europe, Australia, Canada, and the United States, the estimated costs of work-related stress for society ranges between 221.13 million up to 187 billion USD (Hassard et al., 2018). As such, it is important to understand how we can effectively reduce and prevent high stress levels in employees. The current study reports the findings of a field experiment including a 2.5 year intervention implementation project among emergency departments (EDs) in the Netherlands. Next to determining its overall effects, it aims to explore potential moderators related to greater effectiveness.

### What Is Known About Stress Management Interventions

Stress management interventions, programs implemented by organizations to prevent and/or reduce stress and increase employee well-being, are commonly divided in organization-directed (aimed to change the way the work is organized, designed and/or managed) and person-directed (aimed to increase employees’ coping resources) (Holman et al., 2018). The first approach is often preventative and targets the organization as generator of psychosocial hazards (Leka and Cox, 2010). Theoretical background for this type of interventions can be found in the Job-Demands Resources (JD-R) model (Bakker and Demerouti, 2017). The JD-R model states that all job factors can be categorized into either job demands or job resources. Job demands refer to “…those physical, social or organizational aspects of the job that require sustained physical or mental effort and are therefore associated with certain physiological and psychological costs (e.g., exhaustion)” (Demerouti et al., 2001, p. 501). Job resources refer to “those physical, psychological, social, or organizational aspects of the job that may do any of the following: (a) be functional in achieving work goals; (b) reduce job demands at the associated physiological and psychological costs; (c) stimulate personal growth and development” (Demerouti et al., 2001, p. 501). In addition, the model explains the relationship between the working environment and employee well-being by two processes. The health-impairment process states that enduring exposure to high job demands can lead to a depletion of employees’ physical and mental resources and eventually the development of stress-related outcomes (e.g., symptoms of burnout). This energy depletion process is strengthened in the absence and buffered in the presence of adequate job resources (e.g., autonomy and social support). The second process, the motivational process, states that adequate job resources have a motivational role and as such relate to positive outcomes including work engagement and job satisfaction (Bakker and Demerouti, 2017). An organization-directed approach aims to (re)install the balance between job demands and resources, thus preventing stress-related outcomes and increasing employee well-being. The second approach, the person-directed approach, does not aim to change the working environment but instead focusses directly on the (most vulnerable) employees. This approach often includes programs aimed to increase employees’ coping resources (e.g., learning relaxation techniques, enhancing problem solving skills), or providing treatment/rehabilitation for those already experiencing stress-related outcomes (Leka and Cox, 2010).

Regarding successful stress management in organizations, there is general consensus that a multilevel approach including both an organization- and a person-directed intervention, is most effective in reducing as well as preventing stress-related outcomes (Semmer, 2006; Lamontagne et al., 2007; Roberts and Grubb, 2014; McVicar, 2016; Holman et al., 2018). First of all, by targeting the problem at both levels, this approach can reduce the causes of stress whilst at the same time increases employees ability to cope with a demanding working environment (Leka and Cox, 2010; Holman et al., 2018). Furthermore, whilst the person-directed part of the intervention can have an important curative effect (i.e., relieving existing stress-related complaints), the organizational part can work preventative and may also benefit those employees with average well-being (Leka and Cox, 2010). Finally, it has been suggested that within a multilevel approach the person-directed intervention can complement the organization-directed intervention leaving individuals better equipped to deal with changes in the working environment (Lamontagne et al., 2007).

Nevertheless, meta-analyses report moderate to large effects for the person-directed approach whereas the limited number of studies evaluating the organization-directed approach (including multilevel studies) reach little to no effects at all (Van der Klink et al., 2001; Richardson and Rothstein, 2008; Ruotsalainen et al., 2015). Critics argue that the focus on well-being in these studies does not capture the full effect of organization-directed interventions, which primary aim is to optimize the working environment (Semmer, 2006). To understand the effectiveness of these interventions, proximal (job demands and resources) as well as distal effects (well-being) should be studied (Semmer, 2006). Furthermore, the often strict inclusion criterium of a (randomized) controlled design in meta-analyses is not always feasible or even desired to evaluate the effectiveness of the organization-directed approach (Nielsen and Miraglia, 2016; Nielsen and Noblet, 2018). Organizations are dynamic and complex systems and the use of randomized controlled trials to study these type of interventions leads to little external validity; what might work in one organization might not work in another organization (Nielsen and Miraglia, 2016). Instead, scholars advocate the use of a realist approach focusing on how outcomes were achieved (mechanisms or process variables) and under what circumstances (contextual factors) (Nielsen and Miraglia, 2016). The emphasis of this approach lies upon understanding the patterns, in terms of contexts and processes that are related to greater intervention effectiveness (Greenhalgh et al., 2015).

In line with the realist approach, previous research shows that the process by which actions are designed and implemented during an intervention project plays an important role in its overall effectiveness. For example, organizations that design and implement actions that focus on the psychosocial risk factors at hand, are more likely to reach positive results (Nielsen and Randall, 2013; Di Tecco et al., 2020). As such, an effective intervention project includes taking actions that are “fit for purpose” (Leka and Cox, 2010). In addition, clear communication, and employee involvement in determining what kind of actions should be implemented are well known success factors. These processes lead to better understanding in employees on why and how the intervention is supposed to work, increase ownership, and stimulate more positive appraisals toward change (Nielsen and Randall, 2013). In addition, communication and employee involvement results in overall support and active participation of employees in the intervention activities (Nielsen et al., 2010, 2013). Finally, involvement in the project can also have a direct positive impact on employees, including increased job control, social support, role clarity, perceptions of meaningful work and affective well-being (feeling happy and energetic) and feeling less disconnected from work and the organization (Nielsen and Randall, 2009, 2012; Huijs et al., 2019; Schneider et al., 2019).

### The Current Intervention Project

Between 2017 and 2019, a number of emergency departments (EDs) in the Netherlands participated in an intervention implementation project with the aim to reduce psychosocial risk factors at work and improve employee well-being. This project provided an unique opportunity to gain further understanding regarding the effectiveness of stress management interventions over time and to test hypotheses regarding moderating factors that may lead to greater intervention success. Building on lessons learned from previous research, we aim to capture the effect of the intervention project on proximal (job demands and job resources) as well as distal outcomes (well-being). Furthermore, a realist approach was used by not only assessing the outcome of the intervention but also how positive changes during the project occurred including the level of intervening (organization-directed versus multilevel) and the process by which actions were implemented (e.g., communication and employee participation).

**FIGURE 1:**
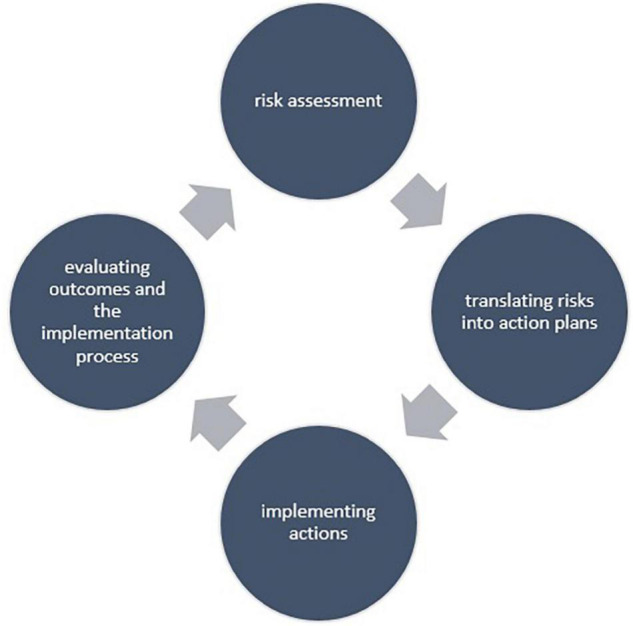
Schematic overview of the main steps in the psychosocial risk management approach “PRIMA.”

The intervention implementation project uses the “psychosocial risk management approach” (PRIMA) (Leka and Cox, 2010). This tool is developed to help organizations to effectively tackle psychosocial risks in their organizations and includes four steps (see Figure 1). The first step, the risk assessment, is meant to determine the most prominent risks within an organization and facilitates the development of fitting actions. In step 2 action plans are developed stating what will be targeted, by whom and within what time frame, and in step 3 these plans are executed. Finally, in step 4, the outcomes of the actions and the process by which they were implemented are evaluated. The last step is important to understand whether the actions reduced psychosocial risks in the organization, and to identify if any new risks appeared. In addition, it creates organizational learning by assessing what worked and what not and if the current approach needs to be adapted.

Although the PRIMA has been applied in various organizations and industries and even translated into interventional frameworks such as the PRIMA-EF (European framework) and the World Health Organization Healthy Workplace Framework, the use of this tool in organizations is still limited (Leka et al., 2015; Bergh et al., 2018). There are a number of potential reasons, including limited understanding of what psychosocial risks entail, and a lack of expertise within the organization to conduct this process (Leka et al., 2015). To overcome this, principles of Participative Action Research (PAR) were integrated. PAR is a type of action research in which researchers and research participants work together to solve practical problems. The approach includes five main principles (Dollard et al., 2008): (1) Important stakeholders are involved in all stages of the project, (2) there is collaboration between researchers and participants in the study, (3) there is empowerment of the research participants to solve self-identified problems, (4) the approach leads to increased local knowledge, and (5) a stronger consensus among employees and management regarding necessary change is developed.

Below the different steps of the intervention implementation project are described. A more detailed overview can be found in the Supplementary Appendix.

#### Preparatory Steps

A multidisciplinary project group was established consisting of two researchers, two project managers from “Stichting IZZ” (a member collective of healthcare workers) and one ED manager. The project group was responsible for the design and execution of the intervention project, and met every 2–3 months to evaluate the process and prepare next steps in the project. As a second preparatory step the scientific literature on psychosocial risks in the ED setting was reviewed. This information was used to develop an occupation-specific questionnaire to measure psychosocial risks and relevant well-being outcomes in the ED setting. Next, the project was presented to EDs in the Netherlands and all EDs were invited to participate. In addition, we aimed to gain management support, an important prerequisite for effective interventions (McVicar et al., 2013; Nielsen and Randall, 2013; Nielsen and Noblet, 2018), by informing ED management about the importance of their commitment to the project and taking actions based on the findings of the risk assessment. Finally, each ED assigned a project manager (often the ED manager) to function as a primary point of contact during the study.

#### Step 1: Conducting a Risk Assessment

At the beginning of the project a risk assessment was conducted to pinpoint the most prominent psychosocial risks to focus on and thus stimulate the development of fitting actions (Leka and Cox, 2010; McVicar et al., 2013; Nielsen and Randall, 2013). In line with recommendations (Leka and Cox, 2010), the risk assessment was performed using a mixed method approach. First, a survey was conducted in January/February 2017 (T1) among the employees of the participating EDs measuring job factors and employee well-being. Participation in the survey was voluntary and upon agreement with the informed consent. Second, semi-structured one-on-one interviews were held with ED employees (five to six employees per ED, randomly chosen) and ED management to gain further understanding of current psychosocial risks. Based upon the risk assessment, each ED received tailored feedback, including an overview of their most prominent psychosocial risks, how to interpret them and a short advice regarding the main points to focus on. Risk factors for all EDs included three job demands: high worktime demands, a high frequency of emotionally demanding situations, and a high frequency of aggression/conflict situations with patients and/or their accompanies, a lack of three job resources namely, limited autonomy, staffing problems and limited recovery opportunities during work time (e.g., breaks), and overall low levels of well-being (e.g., symptoms of burnout).

#### Step 2: Translating Risks Into Action Plans

To support and encourage the EDs to take action, a total of nine inspiration sessions were organized by Stichting IZZ throughout the project. The aim of these inspiration sessions was to enhance the knowledge on stress management and organizational change, and stimulate EDs to exchange ideas and best practices. The sessions were open for ED management as well as employees to attend. Each inspiration session was organized around common problems experienced by the EDs (e.g., “how can I recognize burnout in employees?”, “how can we get psychosocial problems in the ED on the agenda of top management?”, “how can we facilitate regular breaks and stimulate employees to take them?”). In line with PAR principles (Baum et al., 2006; Dollard et al., 2008), the goal of the inspiration sessions was to empower the EDs in designing and implementing their own actions and thus keep control over the intervention project.

#### Step 3: Implementing Interventions

In the current project, EDs were free to choose their own approach in terms of the number and type of actions and how these were implemented. To keep track of what was implemented during the project, project leaders listed all actions taken in their ED to improve job factors and/or employee well-being on a standard form. The form included a description of the action, the start date, the end date (if relevant), the goal, and any comments regarding the action taken. This list was inventoried every 3 to 4 months by the first author followed by a telephone interview to ensure the list was complete and to obtain a better understanding of the actions taken and how the intervention project was evolving (see methods section for examples of implemented actions).

#### Step 4: Evaluating Outcomes and Process Variables

The outcomes and process variables were evaluated half-way the project in June/July 2018 (T2) and at the end of the project in June/July 2019 (T3). For the evaluation a similar mixed-method approach was used as during the risk assessment. First of all, the T1 survey with additional questions regarding how actions were implemented in the ED (e.g., communication and employee participation) was repeated amongst the employees. In addition, we conducted 5–6 interviews with employees in each ED and with ED management. Each ED received an advice report describing any changes in job factors and well-being, and feedback regarding the process by which interventions were implemented. In addition, the overall results were presented to all EDs on one of the inspiration sessions including an advice regarding how to proceed. Based on the results of the T2 survey and the interviews, EDs were strongly advised to improve the process by which the actions were taken (in particular improve communication on, and enhance employee participation in the intervention project) and to also implement person-directed interventions to support employees with severe stress-related complaints. EDs that scored more positively on communication and employee participation during the project (based upon the T2 measurement) and/or had successfully implemented a person-directed intervention were asked to share their approach by means of a presentation, to serve as an inspiration for other EDs.

#### Psychosocial Safety Climate Intervention

During the first year of the project, it became clear that many EDs experienced barriers in implementing actions. Some of these barriers seemed to origin from the limited awareness of hospital top management for the problems experienced by the EDs (mainly regarding the workload, understaffing and consequently overcrowding). As a result, EDs felt they had limited resources (time and financial resources) to make important changes. This was congruent with the suboptimal rating of Psychosocial Safety Climate (PSC) at the baseline risk assessment (T1). PSC concerns an organizational climate referring to prioritization and commitment of all parties within the organization ((top) management, employees, health and safety representatives) to employee well-being (Dollard et al., 2012). Although, up till now, the effects of climate factors on the success of intervention projects has received little research attention, a more favorable PSC has been related to better job factors and employee well-being (Loh et al., 2020). Furthermore, in a pilot study by Dollard (2012) regarding a participative intervention, it was found that in teams with a more favorable PSC, employees attended more workshop sessions, rated the quality of these workshops higher (e.g., ability to discuss issues openly, ability to determine actions to address stress factors) and indicated more progress in the intervention project (e.g., “to what extent are actions from your workgroup action plans being addressed”). Overall, there is good reason to believe that an improvement in PSC will increase the effectiveness of an intervention project. On this basis, all EDs were offered an intervention aimed to optimize PSC within their organization. Eventually, half of the EDs (*k* = 8) participated in this PSC intervention but due to high workload the intervention was first implemented half-way through the project, around T2. Its effects could therefore only be assessed in the final year of the study.

The PSC intervention consisted of three steps. In the first step opinions of employees concerning the most prominent psychosocial risk factors at work were inventoried using a short online questionnaire. As the second step the team discussed the results of this poll to open a dialog on psychosocial risks at work. In a third step, the main points from this dialog were discussed in a meeting between employees and top management of the hospital. All steps were repeated at least three times. This intervention has been studied in various healthcare settings and found to positively impact the overall PSC (Bronkhorst et al., 2018). See Bronkhorst et al. (2018) for a full description of this intervention.

The current project based on PRIMA and PAR principles as described above has a number of assets. First of all, instead of implementing a predefined intervention based upon theoretical problems, PRIMA considers current psychosocial risk factors in the organization. As such, in combination with employee participation and a PAR approach, more fitting interventions can be developed. In addition, organizations are changing entities and new psychosocial risk factors may arise over time. PRIMA is flexible and leaves room to reflect and adjust the current approach if necessary. Furthermore, by giving the EDs an active role in the project, they were empowered to develop their own actions toward stress management. As such, it aims to provide a sustainable solution with regards to effective psychosocial stress management. Finally, from a research perspective it offered the opportunity to test hypotheses in a real-life setting and learn from practical barriers when implementing interventions in an organization.

### Current Study

The research questions addressed by this study are as follows:

1. Is the current intervention project effective in eliciting positive changes in job factors and well-being?
2. What are possible moderators related to more positive changes in job factors and well-being during the intervention project?

As it would be incorrect to keep EDs from taking action to reduce existing psychosocial risk factors during the 2.5 year time frame, it was not feasible to include a suitable control group in the current study. Instead, potential moderators were assessed by comparing the participating EDs retrospectively based upon their approach during the project (multilevel or solely organization-directed) and the process by which they implemented actions (activity during the project, fit of actions to psychosocial risk factors, communication and employee participation). In addition, we compared EDs implementing the PSC intervention during the second half of the project to a self-selected control group (e.g., those EDs not implementing the PSC intervention).

The following hypotheses will be tested:

Hypothesis 1: There is an overall favorable change in job demands, job resources, and employee well-being between T1 and T3.

Hypothesis 2: EDs using a multilevel approach have a more favorable change in employee well-being of employees between T1 and T3, compared to EDs with a solely organization-directed approach.

Hypothesis 3: EDs that are more active (i.e., take more actions during the intervention project) have a more favorable change in job demands, job resources and employee well-being between T1 and T3, compared to EDs that are less active during the project.

Hypothesis 4: EDs that have a greater fit of the actions taken to the identified psychosocial risk factors have a more favorable change in job demands, job resources and employee well-being between T1 and T3, compared to EDs with lower fit of the actions taken.

Hypothesis 5: EDs that score higher on communication about (the process of) actions taken, have a more favorable change in job demands, job resources and employee well-being between T1 and T3, compared to EDs that score lower on communication.

Hypothesis 6: EDs that score higher on employee participation have a more favorable change in job demands, job resources and employee well-being between T1 and T3, compared to EDs that score lower on employee participation.

Hypothesis 7: EDs participating in the PSC intervention around T2 show more positive changes in job demands, job resources and well-being between T2 and T3, compared to EDs not participating in the PSC intervention.

The present study contributes to the literature in multiple ways. First of all, it includes a longitudinal 2.5 year study design examining the effectiveness of an intervention project on proximal (job demands and resources) as well as distal outcomes (employee well-being). It therefore answers to a call by Holman et al. (2018) to gain more insight in the long term effects of stress management interventions. In addition, it adds to the limited amount of studies evaluating an organization-directed or multilevel approach (Richardson and Rothstein, 2008; Ruotsalainen et al., 2015). An approach that theoretically has a lot of potential but still receives limited research attention due to the high amount of necessary (organizational) resources to conduct and evaluate (Heaney and Van Ryn, 1990). Thirdly, it includes a thorough evaluation of potentially moderating factors in the effectiveness of stress management interventions studied in a large group of homogenous organizations and adds to a small body of studies applying the realist approach (Nielsen and Noblet, 2018). Fourth, it concerns a field study and thereby gives a realistic view of stress management approaches used in practice and their effectiveness. Finally, by evaluating the effect of a PSC intervention on job demands, job resources and well-being, it adds to the limited literature on PSC and explores the effect of intervening at the level of the organizational context.

## Materials and Methods

### Setting and Participants

In the fall of 2016 all EDs in the Netherlands were informed about the project. A total of 19 EDs decided to take part, of which 15 EDs participated in all three waves and were included in the current study. This group represented 21% of all EDs in the Netherlands, including four academic hospitals (representing 50% of all academic hospitals in the Netherlands) and four trauma centers (representing 36% of all trauma centers in the Netherlands). Staff demographics and work email addresses were obtained through the Human Resources department of each hospital. Although all employees enlisted in the ED were allowed to participate in the project, for comparison reasons, the current study focused solely on nurses (registered or in training). ED nurses are by far the largest occupational group in the ED. In addition, not all EDs in the Netherlands had physicians enlisted. At baseline (T1) 782 ED nurses were invited to participate (response: *N* = 578, 74%). Due to turnover and hiring of new employees, 831 nurses at T2 (response *N* = 511, 62%) and 861 nurses at T3 (response *N* = 533, 62%) were invited at follow-up surveys. Chi^2^ tests and independent samples *t*-tests showed that respondents at T1 (*N* = 578) worked more hours a week compared to non-responders (*M* = 29.4, *SD* = 6.6 versus *M* = 27.0, *SD* = 10.1). No differences were found in terms of gender, age, occupational role (ED nurse or ED nurse in training), number of years working experience in the ED and whether or not having a supervisory role.

### Measures

#### Employee Well-Being

Well-being was assessed by using a positive (work engagement) as well as a negative (burnout complaints) indicator. This way we would capture both the effect of actions taken to diminish stress-related complaints and to improve employee well-being. To reduce the length of the questionnaire, work engagement was measured with the 3-item version of the Utrecht Work Engagement Scale (UWES-3), which has shown to be a valid and reliable instrument (Schaufeli et al., 2019). Burnout symptoms were measured on its two key dimensions namely emotional exhaustion (8 items) and depersonalization (5 items) (Schaufeli, 2003) with the Dutch version of the Maslach Burnout Inventory-Human Services Survey (MBI-HSS), which is also a reliable and valid questionnaire (Schaufeli and Van Dierendonck, 2000). In both surveys, the items were rated on a 7-point Likert scale from “never” (0) to “daily” (6). The scales had adequate to good internal consistency at each measurement point (ω = 0.77, 0.57, 0.75 for work engagement, ω = 0.89, 0.92, 0.90 for emotional exhaustion and ω = 0.75, 0.82, 0.76 for depersonalization).

#### Job Demands and Resources

A total of five job demands and 11 job resources were assessed which are described in a previous publication on this project (see de Wijn et al., 2022). In the current study we examined job demands and resources that were considered risk factors for all participating EDs based on the risk assessment (T1 survey). Risk factors were identified by comparing the aggregated survey data to available data of nurses from 15 EDs in Belgium (Adriaenssens et al., 2015) and Dutch hospital nurses (Gelsema et al., 2005). Scores on job demands and job resources that were significantly more unfavorable, were identified as risk factors for all EDs. These included three job demands: high worktime demands, a high frequency of emotionally demanding situations, and a high frequency of aggression/conflict situations with patients and/or their accompanies, and three job resources namely, limited autonomy, staffing problems and limited recovery opportunities during work time (e.g., breaks). The questionnaires by which these job demands and resources were assessed, are described in more detail below.

The frequency of *emotionally demanding* (4 items, ω = 79, 0.76, 0.78) and *aggression/conflict situations* (7 items, ω = 0.89, 0.88, 0.89) were measured using an inventory of stressful situations from a study on staff working in organizations providing care for mentally and physically disabled individuals (Bolhuis et al., 2004). An example statement for emotionally demanding situations includes “In my work I am confronted with patients in a hopeless situation.” An example item for aggression/conflict situations includes “In my work I am confronted with patients and/or accompanies who are physically aggressive.” All statements were answered on a 7-point Likert scale from “never” (1) to “daily” (7).

*Worktime demands*, *autonomy* and *staffing* were measured with the nurse version of the Leiden Quality of Work Questionnaire (LQWQ-n) (Maes et al., 1999; Gelsema et al., 2005). The LQWQ-n is an occupation specific questionnaire which has shown to be a reliable instrument in several studies (Adriaenssens et al., 2012; Van Bogaert et al., 2014). An example item for worktime demands includes “I must care for too many patients at once,” for autonomy “I have the opportunity to make my own decisions at work” and for staffing “There are enough nurses on my ward to provide good care.” Statements were answered on a 4-point Likert scale from “entirely disagree” (1) to “entirely agree” (4). Worktime demands (5 items, ω = 0.72, 0.71, 0.76), and staffing (4 items, ω = 0.79, 0.76, 0.78) had good internal consistency. The internal consistency of autonomy was modest (ω = 0.61, 0.60, 0.67). Removing one item for autonomy did not lead to greater internal consistency and thus the original 4-item scale was used. In addition, it has been argued that for small scales (e.g., less than ten items) it is more appropriate to assess the internal consistency of the scale by the mean of the inter-item correlations (Pallant, 2011, p. 97). The average of the inter-item correlations was 0.268 which is within the suggested optimal range (0.20 to 0.40) (Briggs and Cheek, 1986).

*Recovery opportunities during worktime* was measured using a self-developed questionnaire consisting of four statements 1. “If I want to, I can leave my workplace for a short while,” 2. “I can have a chat during my work,” 3. “During my shift, I regularly have to skip breaks” (reversed), 4. “During my breaks, I must remain available for urgent cases” (reversed), which were answered on a 4-point Likert scale from “never” (1) to “always” (4). The internal consistency was modest (ω = 0.61, 0.58, 0.57). Removing one item from the scale did not lead to higher internal consistency. As such, the original 4-item scale was used. The average of the inter-item correlations was 0.262, which is within recommendations (0.20 to 0.40) (Briggs and Cheek, 1986; Pallant, 2011, p. 97). Regarding face validity, all items concerned opportunities to mentally or physically distance from work during worktime (or the opposite in the reversed items). To assess construct validity, a principal component analysis was conducted on the four items of within worktime recovery. Both Kaiser’s criterion of an eigenvalue over one and the inflection of the scree plot justified retaining one factor, which explained 44.9% of the variance. Factor loadings ranged from 0.57 to 0.74, which is above the recommended threshold of 0.40 (Lloret et al., 2014).

#### Psychosocial Safety Climate

Psychosocial Safety Climate (PSC) was measured using the adapted version of the PSC-12 scale (Hall et al., 2010; Bronkhorst, 2015). This scale consists of five factors, 1. Priority by top management for psychosocial health and safety, 2. commitment by direct management to maintain/increase psychosocial health and safety, 3. participation of all stakeholders [e.g., (top) management, employees, human resources, occupational health representatives] within the organization to reduce psychosocial risks at work, 4. communication within the organization on psychosocial health and safety and 5. the group norm toward psychosocial health and safety. Each factor consisted of three statements answered on a 5-point Likert scale from “totally disagree” (1) to “totally agree” (5). The full scale had excellent internal consistency (ω = 0.93, 0.93, 0.93).

#### Moderators

*The level of intervening* was based upon the list of actions as provided by the project leaders. EDs were divided into two groups: one group using a solely organization-directed approach (*k* = 5) and one group including an organization-directed as well as a person-directed approach (i.e., a multilevel approach) (*k* = 10). None of the EDs had a solely person-directed approach.

*Activity* reflects the number of actions by the ED during the intervention project also based upon the list of actions. Only actions that were taken between T1 and T3 and fitted the definition of a stress management intervention “… any activity, or program, or opportunity initiated by an organization, which focuses on reducing the presence of work-related stressors or on assisting individuals to minimize the negative outcomes of exposure to these stressors” (Ivancevich et al., 1990, p. 252), were included. To avoid double counting, preparatory actions (e.g., setting up a workgroup) were omitted. Some examples of actions taken during the intervention project included: expanding the number of ED nurse trainees and supporting staff, having medical specialists working shifts on the ED during peak hours, optimizing patient flow by dividing the department in a low care and high care unit, taking security measures (e.g., doors that can only be opened by staff), psychoeducation on burnout symptoms, coaching to improve communication within the team, changing work shifts to ensure the possibility of taking breaks, and the introduction of self-rostering. Based upon the follow up telephone interviews with project managers, it became clear that although the assessment of activity provided a good estimate, it was not a perfect count of the actual activity in the EDs. As such, it was decided to use a median split to differentiate between EDs with lower activity (<17 actions taken, *k* = 7) and EDs with higher activity (≥17 actions taken, *k* = 8).

*Fit* of actions was also based upon the inventory. In line with recommendations (Nielsen and Randall, 2013) we aimed to assess the fit by comparing the identified risks on the risk assessment to the goals of the actions listed. However, it appeared that project leaders had difficulties stating the goals for the actions taken in de ED, leaving it either blank or reporting distal goals (e.g., to improve employee well-being). Therefore, an alternative approach was used. For each of the six identified psychosocial risk factors at T1 (three job demands and three job resources), the first author screened the list of actions to evaluate whether any of the actions taken by the ED targeted this risk factor (e.g., a fitting action). Due to the high prevalence of stress-related outcomes (e.g., burnout complaints) as identified on the T1 measurement, we also labeled actions directly focused on employee well-being (e.g., coaching or meetings with a psychologist) as fitting actions. In case it was unclear whether an action could be regarded as “fitting” to any of these risk factors, it was discussed with the second author of this paper until consensus was reached. Finally, a third researcher not involved in the current project was asked to code the actions of four randomly chosen EDs. The percentage overlap of the coding by this researcher and the coding of the first author was 93%. In addition, Cohen’s Kappa was 0.83 (*p* < *0.001*), which is considered a strong inter-rater agreement (0.80–0.90) (McHugh, 2012). Fit was calculated for each ED by dividing the number of risk factors taken action upon by the total number of risk factors. As such, a 100% fit indicates that actions had been taken for all of the seven risk factors (six demands and resources, and employee well-being in general). In line with activity, a median split was used to differentiate between EDs with lower (<71%, *k* = 7) and higher (≥71%, *k* = 8) fit.

*Communication* and *employee participation* were measured on the T2 and T3 surveys. The items were based on the Intervention Process Measure (Nielsen and Randall, 2009). The scale was introduced by giving a general description on actions that might have been taken in the ED in the past year. Next, *communication* was measured with one item; “I am informed on the progress of such actions/interventions” and *employee participation* was measured with three items: 1. “I am involved in developing/implementing such actions,” 2. “As an employee, I feel (partly) responsible for the implementation of such actions,” and 3. “I have the opportunity to comment on such actions before they are implemented.” All statements were answered on a 7 point Likert scale from “not at all” (1) to “a very high degree” (7). Participation had good internal consistency (ω = 0.82, 0.86). The average on communication and on employee participation from the T2 and T3 measurements, was used to indicate an overall score on communication and participation during the whole project. The data was aggregated to the ED level and a medium split was used to divide between EDs that scored lower (<3.95, *k* = 7) and higher on communication (≥3.95, *k* = 8), and EDs that scored lower (<3.68, *k* = 8) and higher on employee participation (≥3.69, *k* = 7). A median split was used as we expected that the moderating effect of communication and participation would reflect a threshold effect, rather than a dose response relationship. Thus, we expected a different effect over time between EDs that communicated more versus those that communicate less and between EDs that involved their employees more in the process versus those that did so less.

*Psychosocial Safety Climate Intervention.* For the moderation analyses we distinguished EDs that implemented the PSC intervention around T2 (*k* = 8) and EDs that did not (*k* = 7).

### Statistical Analyses

The data had a three-level hierarchical structure: Time points (level 1) were nested within employees (level 2) and employees were nested within EDs (level 3). To account for the nested structure we performed linear mixed-model analyses using the lme4 package in *R* (version 1.1–26; Bates et al., 2015). For all analyses, a *p*-value of 0.05 was used to indicate significant differences. First we aimed to assess the effect of the intervention implementation project over time. Nine linear mixed models were fitted (one for each of the dependent variables) with a random intercept for ED and a random intercept for nurse, and time as a fixed effect. Time was coded as a categorical variable, with T1 as the reference category, because we did not expect change would necessarily follow a linear pattern over time. In case a significant effect of time was found, *post hoc* pairwise comparisons were performed using the Tukey Method to adjust for multiple testing. This way we could identify between what time points (T1, T2, and T3) there was a significant change in the dependent variable over time.

Next, it was assessed whether the change over time differed for EDs depending on the level of intervening (multilevel or organization-directed), implementation process (activity, fit, communication, employee participation) and whether or not partaking in a PSC intervention between T2 and T3. To study this, a series of linear mixed models were fitted, one for each combination of potential moderator and dependent variable. Again, we included a random intercept for ED and nurse to adjust for the nested structure. We included the interaction between time and the potential moderator under study as a fixed effect. In case of a significant interaction effect, *post hoc* pairwise comparisons using the Tukey Method were performed for each level of the moderator to test which time points differed significantly. In addition, significant interaction effects were plotted to support interpretation of the effect. An advantage of mixed-model analyses (compared to for example MANOVA) is that each level 2 unit is allowed to have a different number of observations at level 1. Thus, all nurses with data on at least one time point can be included in the analyses. However, because we are interested in change over time, we opted to include only nurses with data on at least two out of the three time points. Because some nurses completed only a subset of assessments at some time points, the analyses include 483 to 521 nurses depending on the dependent variable under study.

## Results

### Preliminary Analyses

All assumptions of performing linear mixed-model analyses were met with the exception of the homogeneity of variances assumption. Histograms showed that aggression/conflict situations, emotional exhaustion and depersonalization were skewed to the left, whereas work engagement was skewed to the right. We performed a log(x) transformation for aggression/conflict situations, a log (x + 1) transformation for emotional exhaustion and depersonalization, and a x^2 transformation for work engagement resulting in increased normality of the residuals and improved homogeneity. Next, we calculated the intraclass correlation coefficient (ICC) for each of the dependent variables to assess how much of the variability in the dependent variable was due the ED level. This resulted in a ICC(1) of 0.17 for worktime demands, 0.07 for aggression/conflict situations, 0.04 for emotional demanding situations, 0.02 for autonomy, 0.19 for staffing, 0.13 for within worktime recovery, 0.08 for work engagement, 0.07 for emotional exhaustion and 0.06 for depersonalization. As shown by Musca et al., 2011 an ICC of 0.01 can already lead to increased Type I error. As such, these results confirm the decision of performing linear mixed-model analyses to correct for the nested structure of the data.

### Changes in Job Demands, Resources and Well-Being Over Time

First of all, we assessed whether the project resulted in overall improvements in job demands, job resources and employee well-being over time (hypothesis 1). The results of these analyses are presented in Table 1. We found significant changes in all job demands and all job resources, with the largest effects for staffing (η^2^ = 0.07) and worktime demands (η^2^ = 0.06). *Post hoc* comparisons showed that between T1 and T3 worktime demands, aggression/conflict situations and emotionally demanding situations decreased, whilst staffing levels and within worktime recovery increased. Autonomy only improved in the second half of the project (T2–T3), but not overall (T1–T3). In addition, the results showed that most of the positive changes in job factors occurred during the second half of the project (between T2–T3), with the exception of aggression/conflict situations. Finally, significant changes over time were found for all indicators of well-being (work engagement, emotional exhaustion and depersonalization). However, *post hoc* comparisons showed that work engagement decreased over the course of the project (T1–T3). Indicators of burnout (emotional exhaustion and depersonalization) showed a small but significant increase during the second half of the project (T2–T3) but remained stable when considering the whole timeframe (T1–T3).

**TABLE 1:** Changes over time in job demands, job resources and well-being during the intervention project.

								*Post hoc* pairwise comparisons					
Dependent variable	*F*	Numdf	Dendf	*p-value*	Eta^2^	[95% CI]	*N*	timepoint	*E* *M*	SE	df	*t*	*p-value*
**Job demands**													
Worktime demands	26.14	2	837	0.00	0.06	[0.03–0.09]	521	T1–T2	–0.01	0.02	834	–0.35	0.935
								T2–T3	–0.13	0.02	841	–6.22	< 0.001
								T1–T3	–0.14	0.02	856	–6.40	< 0.001
Aggression^a^	6.07	2	762	0.00	0.02	[0.00–0.03]	483	T1–T2	–0.04	0.01	757	–3.35	0.003
								T2–T3	0.01	0.01	764	0.71	0.757
								T1–T3	–0.04	0.01	771	–2.51	0.033
Emotional demands	13.10	2	770	0.00	0.03	[0.01–0.06]	483	T1–T2	–0.09	0.05	761	–1.89	0.144
								T2–T3	–0.17	0.05	768	–3.33	0.003
								T1–T3	–0.26	0.05	776	–5.06	< 0.001
**Job resources**													
Autonomy	4.03	2	835	0.02	0.01	[0.00–0.03]	521	T1–T2	–0.03	0.02	830	–1.67	0.216
								T2–T3	0.05	0.02	837	2.81	0.014
								T1–T3	0.02	0.02	851	1.13	0.496
Staffing	33.17	2	828	0.00	0.07	[0.05–0.11]	503	T1–T2	–0.11	0.03	824	–4.03	< 0.001
								T2–T3	0.23	0.03	833	8.14	< 0.001
								T1–T3	0.12	0.03	849	4.06	< 0.001
Within worktime recovery	21.94	2	844	0.00	0.05	[0.03–0.08]	521	T1–T2	0.02	0.02	837	0.85	0.671
								T2–T3	0.11	0.02	845	5.41	< 0.001
								T1–T3	0.13	0.02	860	6.10	< 0.001
**Well-being**													
Work engagement*^b^*	56.33	2	790	0.00	0.12	[0.09–0.16]	494	T1–T2	–4.11	0.42	784	–9.70	< 0.001
								T2–T3	0.31	0.43	790	0.72	0.751
								T1–T3	–3.80	0.44	801	–8.61	< 0.001
Emotional exhaustion*^c^*	4.09	2	780	0.02	0.01	[0.00–0.02]	495	T1–T2	–0.02	0.02	776	–0.94	0.613
								T2–T3	0.05	0.02	781	2.83	0.013
								T1–T3	0.03	0.02	789	1.85	0.153
Depersonalization*^c^*	7.37	2	778	0.00	0.02	[0.01–0.03]	495	T1–T2	–0.06	0.02	776	–3.49	0.002
								T2–T3	0.05	0.02	781	3.04	0.007
								T1–T3	–0.01	0.02	789	–0.38	0.925

*T1 = 2017, T2 = 2018, T3 = 2019; numDF, df numerator; denDF, df denominator; CI, Confidence Interval; EM, estimated mean difference.*

*Post hoc pairwise comparisons p-value adjustment: Tukey method for comparing a family of 3 estimates.*

*^a^transformed variable: log(x).*

*^b^transformed variable: (x^2).*

*^c^transformed variable: log(x + 1).*

### Influence of the Level of Intervening

The results of the moderation analyses and *post hoc* pairwise comparisons for significant group*time interactions effects are displayed in Tables 2, 3.

**TABLE 2:** Changes over time in job demands, job resources and well-being depending on the intervention level (multilevel or organization-directed), activity (lower versus higher), fit of actions (lower versus higher), communication (less versus more) and employee participation (less versus more) in the intervention project.

	Intervention level Multi (*k* = 10) versus OD (*k* = 5) group*time				Activity Lower (*k* = 7) versus higher (*k* = 8) group*time				Fit Lower (*k* = 7) versus higher (*k* = 8) group*time				Communication Less (*k* = 7) versus more (*k* = 8) group*time				Employee participation Less (*k* = 8) versus more (*k* = 7) group*time			
	*F*	numDF	denDF	*p-value*	*F*	numDF	denDF	*p-value*	*F*	numDF	denDF	*p-value*	*F*	numDF	denDF	*p-value*	*F*	numDF	denDF	*p-value*
**Job Demands**																				
WTD	NA	NA	NA	NA	0.03	2	834	0.968	0.45	2	836	0.639	3.14	2	835	0.044	2.36	2	835	0.095
AGGR^a^	NA	NA	NA	NA	0.22	2	756	0.805	1.56	2	758	0.211	0.58	2	757	0.559	1.44	2	757	0.238
EMOD	NA	NA	NA	NA	0.79	2	768	0.452	0.05	2	769	0.947	1.11	2	768	0.331	0.59	2	767	0.553
**Job Resources**																				
AUT	NA	NA	NA	NA	0.94	2	831	0.389	0.35	2	834	0.705	4.64	2	834	0.010	2.57	2	833	0.077
STAFF	NA	NA	NA	NA	3.55	2	824	0.029	8.21	2	827	<0.001	4.02	2	826	0.018	5.13	2	825	0.006
RECOV	NA	NA	NA	NA	1.43	2	840	0.239	2.61	2	842	0.074	1.44	2	842	0.236	1.64	2	841	0.194
**Well-being**																				
WE^b^	0.01	2	787	0.994	0.19	2	787	0.831	0.67	2	789	0.511	0.04	2	789	0.957	0.61	2	788	0.544
EE^c^	7.93	2	777	<0.001	3.14	2	777	0.044	0.73	2	779	0.480	2.49	2	778	0.084	4.14	2	778	0.016
DP^c^	5.14	2	775	0.006	0.54	2	776	0.584	1.46	2	777	0.232	0.57	2	776	0.567	0.89	2	776	0.409

*WTD, worktime demands; AGGR, aggression/conflict situations; EMOD, emotional demanding situations; AUT, autonomy; STAFF, staffing; RECOV, opportunities for within worktime recovery; ENG, work engagement; EE, emotional exhaustion; DP, depersonalization; Multi, multilevel; OD, organization-directed; k, number of emergency departments; numDF, df numerator; denDF, df denominator; NA, not applicable.*

*^a^transformed variable: log(x).*

*^b^transformed variable: (x^2).*

*^c^transformed variable: log(x + 1).*

**TABLE 3:** *Post hoc* pairwise comparisons for significant group*time effects.

	contrast	Estimated mean difference	*SE*	*df*	*t ratio*	*p-*value	contrast	Estimated mean difference	*SE*	*df*	*t ratio*	*p-*value
	**Level = organization-directed (*k* = 5)**						**Level = multilevel (*k* = 10)**					
EE^a^	T1–T2	–0.09	0.03	773	–3.11	0.006	T1–T2	–0.03	0.02	775	–1.19	0.457
	T2–T3	0.13	0.03	778	4.62	< 0.001	T2–T3	0.00	0.02	779	–0.04	0.999
	T1–T3	0.04	0.03	780	1.57	0.261	T1–T3	–0.03	0.02	791	–1.17	0.471
DP^a^	T1–T2	–0.13	0.03	773	–4.50	< 0.001	T1–T2	–0.02	0.02	775	–0.94	0.614
	T2–T3	0.11	0.03	778	3.86	< 0.001	T2–T3	0.02	0.02	779	0.90	0.644
	T1–T3	–0.02	0.03	780	–0.54	0.852	T1–T3	–0.00	0.02	790	–0.03	0.999
	**Activity = lower (*k* = 7)**						**Activity = higher (*k* = 8)**					
STAFF	T1–T2	–0.04	0.04	808	–0.99	0.586	T1–T2	–0.16	0.04	833	–4.45	< 0.001
	T2–T3	0.15	0.04	836	3.33	0.003	T2–T3	0.28	0.04	827	7.85	< 0.001
	T1–T3	0.11	0.04	840	2.37	0.047	T1–T3	0.12	0.04	852	3.22	0.004
EE^a^	T1–T2	–0.07	0.03	767	–2.47	0.036	T1–T2	0.02	0.02	780	0.89	0.650
	T2–T3	0.08	0.03	780	3.03	0.007	T2–T3	0.03	0.02	778	1.14	0.493
	T1–T3	0.02	0.03	783	0.68	0.775	T1–T3	0.05	0.02	790	1.96	0.124
	**Fit = lower (*k* = 7)**						**Fit = higher (*k* = 8)**					
STAFF	T1–T2	–0.07	0.04	819	–1.90	0.140	T1–T2	–0.14	0.04	826	–3.62	0.001
	T2–T3	0.11	0.04	844	2.76	0.016	T2–T3	0.34	0.04	818	8.70	< 0.001
	T1–T3	0.04	0.04	835	0.95	0.609	T1–T3	0.19	0.04	859	4.73	< 0.001
	**Communication = lower (*k* = 7)**						**Communication = higher (*k* = 8)**					
WTD	T1–T2	0.04	0.03	833	1.23	0.438	T1–T2	–0.05	0.03	831	–1.73	0.195
	T2–T3	–0.17	0.03	843	–5.96	< 0.001	T2–T3	–0.08	0.03	836	–2.85	0.012
	T1–T3	–0.14	0.03	858	–4.63	< 0.001	T1–T3	–0.13	0.03	850	–4.45	< 0.001
AUT	T1–T2	–0.05	0.02	831	–2.14	0.083	T1–T2	–0.01	0.02	827	–0.27	0.962
	T2–T3	0.02	0.02	838	0.70	0.765	T2–T3	0.08	0.02	832	3.26	0.010
	T1–T3	–0.03	0.03	853	–1.37	0.359	T1–T3	0.07	0.03	845	2.93	0.010
STAFF	T1–T2	–0.17	0.04	823	–4.43	< 0.001	T1–T2	–0.05	0.04	820	–1.30	0.394
	T2–T3	0.30	0.04	835	7.60	< 0.001	T2–T3	0.16	0.04	827	3.95	< 0.001
	T1–T3	0.13	0.04	851	3.15	0.005	T1–T3	0.11	0.04	842	2.61	0.025
	**Employee participation = lower (*k* = 8)**						**Employee participation = higher (*k* = 7)**					
STAFF	T1–T2	–0.10	0.04	829	–2.66	0.022	T1–T2	–0.13	0.04	812	–3.09	0.006
	T2–T3	0.15	0.04	832	4.14	< 0.001	T2–T3	0.33	0.04	828	7.69	< 0.001
	T1–T3	0.06	0.04	847	1.46	0.312	T1–T3	0.20	0.04	846	4.57	< 0.001
EE^a^	T1–T2	–0.03	0.02	778	–1.11	0.506	T1–T2	–0.01	0.03	770	–0.20	0.978
	T2–T3	0.09	0.02	780	3.96	< 0.001	T2–T3	–0.01	0.03	778	–0.20	0.979
	T1–T3	0.07	0.02	787	2.79	0.015	T1–T3	–0.01	0.03	786	–0.38	0.924

*T1 = 2017, T2 = 2018, T3 = 2019; WTD, worktime demands; AGGR, aggression/conflict situations; EMOD, emotional demanding situations; AUT, autonomy; STAFF, staffing; RECOV, opportunities for within worktime recovery; ENG, work engagement; EE, emotional exhaustion; DP, depersonalization; k, number of emergency departments; p-value adjustment: Tukey method for comparing a family of 3 estimates.*

*^a^transformed variable: log(x + 1).*

**FIGURE 2:**
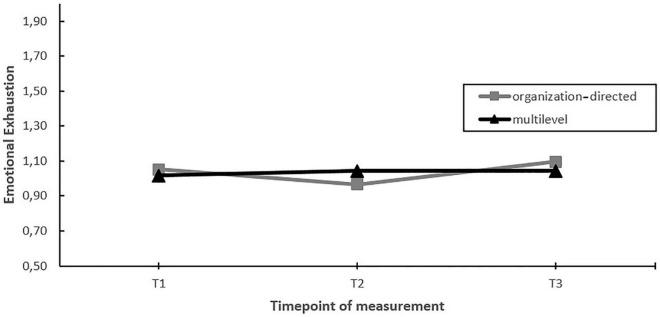
Moderation effect of emergency departments with a multilevel approach versus emergency departments with a solely organization-directed approach toward stress management on changes in emotional exhaustion over time. T1 = 2017, T2 = 2018, T3 = 2019.

**FIGURE 3:**
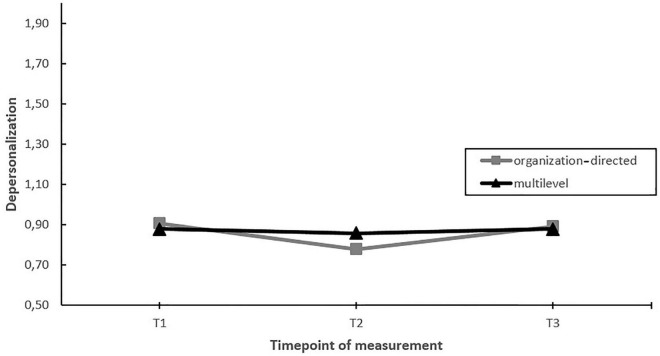
Moderation effect of emergency departments with a multilevel approach versus emergency departments with a solely organization-directed approach toward stress management on changes in depersonalization over time. T1 = 2017, T2 = 2018, T3 = 2019.

First, we assessed whether EDs with a multilevel approach toward stress management yielded greater improvements in employee well-being (work engagement, emotional exhaustion and depersonalization) compared to EDs using an solely organization-directed approach (hypothesis 2). The findings indicated a moderating effect of the level of intervening on burnout symptoms (emotional exhaustion and depersonalization) over time (see Table 2). Nevertheless, *post hoc* pairwise comparisons showed that the moderating effect was the result of differential changes during the project (i.e., changes between T1–T2 or T2–T3), but not when considering the whole timeframe (T1–T3) (see Figures 2, 3).

### Influence of Activity

**FIGURE 4:**
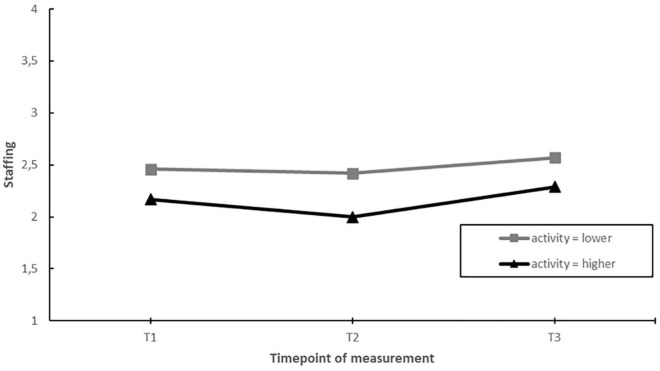
Moderation effect of emergency departments with higher activity (more actions implemented) compared to emergency departments with lower activity during the intervention project on changes in staffing over time. T1 = 2017, T2 = 2018, T3 = 2019.

**FIGURE 5:**
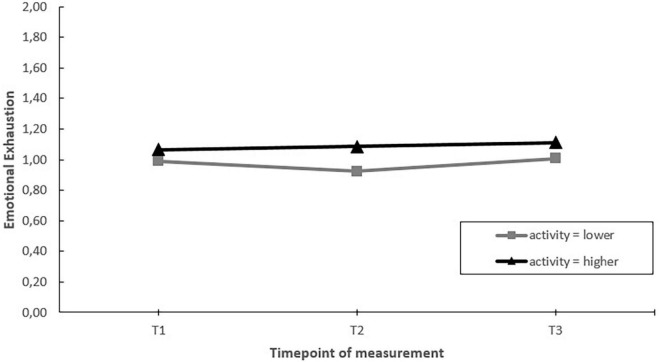
Moderation effect of emergency departments with higher activity (more actions implemented) compared to emergency departments with lower activity during the intervention project on changes in emotional exhaustion over time. T1 = 2017, T2 = 2018, T3 = 2019.

Second, we assessed whether EDs implementing more actions during the project yielded greater improvements in job factors and employee well-being over time, compared to EDs that were less active during the project (hypothesis 3). The results showed that activity had a significant moderating effect on staffing levels and emotional exhaustion over time. Nevertheless, *post hoc* pairwise comparisons showed that the moderating effect was the result of differential changes during the project (i.e., changes between T1–T2 or T2–T3), but not when considering the whole timeframe (T1–T3) (see Figures 4, 5).

### Influence of Fit to Psychosocial Risk Factors

**FIGURE 6:**
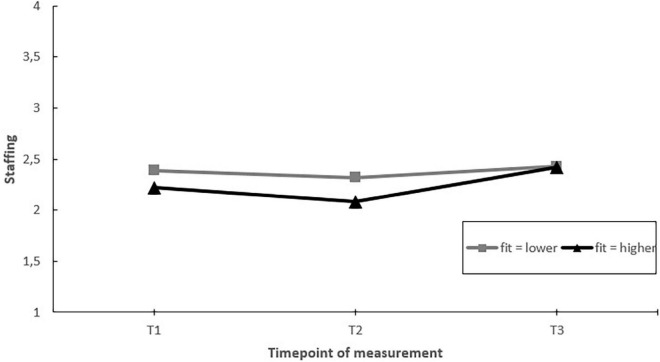
Moderation effect of emergency departments with better fit of the implemented actions to the psychosocial risk factors versus emergency departments with lower fit of actions implemented during the project on changes in staffing over time. T1 = 2017, T2 = 2018, T3 = 2019.

Third, we assessed whether EDs implementing more fitting actions to the identified psychosocial risk factors had greater improvements in job factors and employee well-being during the project, in comparison to EDs implementing less fitting actions (hypothesis 4). The results showed a significant moderating effect of fit on perceived staffing levels over time. EDs implementing more fitting actions showed a significant increase in staffing levels when comparing the T1 and T3 measurements. In comparison, in EDs implementing less fitting actions, no significant changes in staffing levels were found when comparing the T1 and T3 measurements. The moderating effect mainly occurred due to changes in the second half of the project (see Figure 6).

### Influence of Communication

**FIGURE 7:**
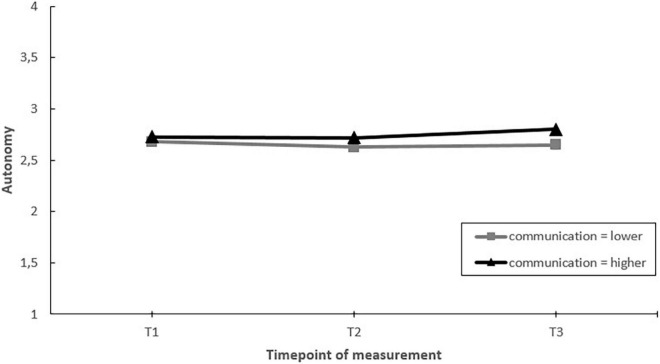
Moderation effect of emergency departments with higher levels of communication versus emergency departments with lower levels of communication during the intervention project on changes in autonomy over time. T1 = 2017, T2 = 2018, T3 = 2019.

**FIGURE 8:**
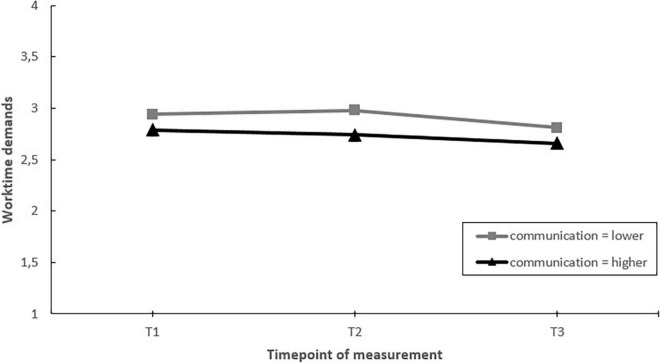
Moderation effect of emergency departments with higher levels of communication versus emergency departments with lower levels of communication during the intervention project on changes in worktime demands over time. T1 = 2017, T2 = 2018, T3 = 2019.

**FIGURE 9:**
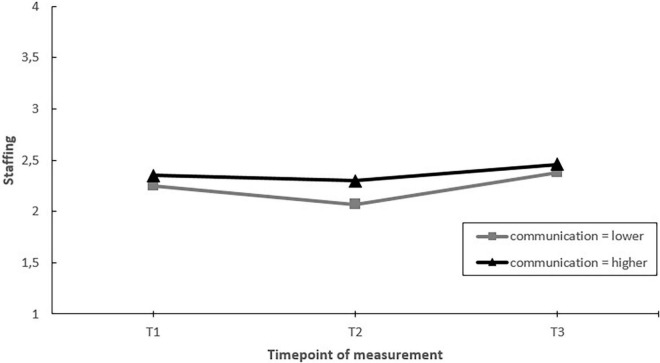
Moderation effect of emergency departments with higher levels of communication versus emergency departments with lower levels of communication during the intervention project on changes in staffing over time. T1 = 2017, T2 = 2018, T3 = 2019.

Next, we assessed whether EDs that communicated more on the project toward employees had greater improvements in job factors, job resources and well-being, than EDs that communicated less (hypothesis 5). The results showed significant moderating effects of communication on changes in worktime demands, autonomy, and staffing over time. *Post hoc* pairwise comparisons showed that in EDs communicating more, autonomy increased over the course of the project (T1–T3). In contrast, no change in autonomy was found in EDs that communicated less (Figure 7). Regarding worktime demands and staffing, *post hoc* pairwise comparisons showed that the moderating effect was the result of differential changes during the project (i.e., changes between T1–T2 or T2–T3), but not when considering the whole timeframe (T1–T3) (Figures 8, 9).

### Influence of Employee Participation

**FIGURE 10:**
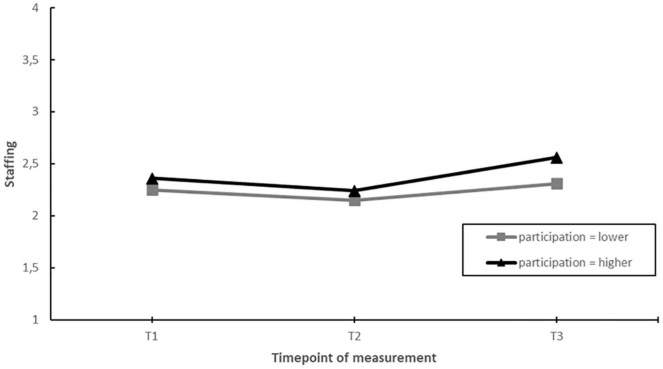
Moderation effect of emergency departments with higher levels of employee participation versus emergency departments with lower levels of employee participation during the intervention project on changes in staffing over time. T1 = 2017, T2 = 2018, T3 = 2019.

**FIGURE 11:**
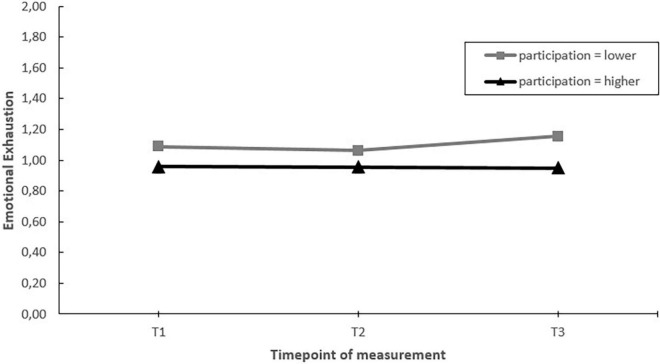
Moderation effect of emergency departments with higher levels of employee participation versus emergency departments with lower levels of employee participation during the intervention project on changes in emotional exhaustion over time. T1 = 2017, T2 = 2018, T3 = 2019.

We assessed whether those EDs that involved their employees more in designing and implementing actions during the project showed greater improvements in job demands, job resources and employee well-being than those that involved their employees less (hypothesis 6). Moderating effects were found for staffing and emotional exhaustion. *Post hoc* pairwise comparisons showed that EDs with more employee participation, had a greater increase in perceived staffing levels over the course of the project (T1–T3). In addition, EDs with more employee involvement had stable levels of emotional exhaustion, whereas emotional exhaustion increased in those EDs with less employee participation. These moderating effects mainly occurred in the second half of the project (T2–T3) (see Figures 10, 11).

### Influence of a Psychosocial Safety Climate Intervention

Finally, we assessed whether the EDs that participated in the Psychosocial Safety Climate (PSC) intervention around T2 had more positive changes in job demands, job resources and well-being between T2 and T3, compared to EDs not participating in this intervention (hypothesis 7). First, we checked whether the intervention was indeed effective in increasing PSC in the participating EDs. A linear mixed-model analysis was performed with a random intercept for the EDs and the nurses to adjust for the nested structure of the data and a group*time interaction as a fixed effect. Levels of PSC at T2 were similar for those EDs participating and those not participating in the PSC intervention. In addition, the results showed a significant interaction effect of the PSC intervention on PSC levels over time between T2 and T3 [*F*(1,474) = 14.72, *p* < *0.001*]. *Post hoc* paired comparisons showed that PSC increased in those EDs participating in the PSC intervention [estimated mean difference = 0.235, *t*(504) = 5.716, *p* < *0.001*] and remained stable in those EDs not participating in the PSC intervention [estimated mean difference = −0.003, *t*(471) = −0.061, *p* = 0.951]. As such, we can conclude that the intervention was effective in increasing PSC in the participating EDs.

Linear mixed-model analyses for each of the job demands, job resources and well-being indicators showed no significant moderating effect of (non)involvement in the PSC intervention (see Table 4).

**TABLE 4:** The moderating effect of implementing a psychosocial safety climate intervention on changes in job demands, job resources and employee well-being between T2 and T3.

	PSC intervention yes (*k* = 8) versus no (*k* = 7) group*time			
	*F*	numDF	denDF	*p-value*
**Job demands**				
Worktime demands	3.82	1	355	0.051
Aggression^a^	2.67	1	325	0.103
Emotional demands	0.67	1	325	0.413
**Job resources**				
Autonomy	0.22	1	355	0.639
Staffing	0.85	1	347	0.358
Within worktime recovery	0.02	1	355	0.894
**Well-being**				
Work Engagement^b^	0.19	1	337	0.660
Emotional Exhaustion^c^	1.03	1	338	0.312
Depersonalization^c^	1.05	1	338	0.306

*PSC, Psychosocial Safety Climate; k, number of emergency departments; numDF, df numerator; denDF, df denominator.*

*^a^transformed variable: log(x).*

*^b^transformed variable: (x^2).*

*^c^transformed variable: log(x + 1).*

## Discussion

The current study reports on the results of a 2.5 year intervention implementation project in emergency departments (EDs) in the Netherlands. The project was based on the “psychosocial risk management approach” (PRIMA) including cycles of assessing psychosocial risks, implementing actions, evaluating the implementation process and outcomes and adjusting the approach if needed. In addition, principles of participative action research (PAR) including an active role of participants throughout the project were integrated: EDs were empowered to design and implemented their own actions during the project. Finally, based upon the halfway evaluation an intervention to increase Psychosocial Safety Climate (PSC) was offered and half of the EDs took part. To pinpoint factors related to greater effectiveness of the project, potential moderators including the level of intervening (an organization-directed or multilevel approach), process variables (the number and fit of actions, communication and employee participation) and taking part in the PSC intervention were assessed. Overall, several favorable effects on job demands and job resources were present. Worktime demands, the frequency of aggression/conflict situations and emotional demands decreased over the course of the project, whilst perceived staffing levels and within worktime recovery increased. Autonomy showed an increase during the second half of the project (T2–T3), but not when considering the entire timeframe (T1–T3). Nevertheless, no beneficial effects were found for employee well-being: Work engagement decreased during the project, whilst no changes were found in burnout levels considering the entire timeframe of the project (T1 versus T3). Moderation analyses showed that those EDs that took more fitting actions to the identified psychosocial risks, that communicated better and/or involved their employees more in the intervention project, showed more favorable changes over time. In contrast, no differences were found with regard to the level of intervening (i.e., multilevel or a solely organization-directed approach) or activity during the project (i.e., less or more actions taken) considering the entire timeframe of the project (T1 versus T3). Finally, although the effects of implementing a PSC intervention could only be assessed for the latter half of the project, it did improve PSC in the participating EDs, but no effects on job factors or well-being were found.

### Changes in Job Demands, Job Resources, and Well-Being

In line with our expectations favorable changes occurred over the course of the project, including a decrease of job demands and an increase in job resources with the exception of autonomy. Autonomy showed a significant increase during the second half of the project, but not when considering the entire timeframe (T1 versus T3). A potential explanation for the overall unchanged levels of autonomy is that little actions were taken that focused on increasing this resource. Another reason refers to the moderate reliability of autonomy which makes it possible that slight changes in autonomy were not detected by our measure. Still, it should be noted that according to the moderation analyses job autonomy did increase in those EDs that communicated more on the (progress of) the intervention project. This suggests that even without specific actions, job autonomy can be increased by keeping employees informed on the progress of the intervention project and any upcoming changes, which is in line with findings of previous studies (Nielsen and Randall, 2012; Nielsen and Noblet, 2018).

Against what would be expected based on the motivational pathway of the JD-R model, we found an improvement of most job resources whilst work engagement decreased. There are a number of potential reasons why work engagement diminished during the project. First of all, the awareness the project created for psychosocial risk factors might have shifted the attention of employees to the negative aspects of their work. Second, symptoms of burnout, a stress-related outcome which was highly prevalent amongst ED nurses, can over time lead to reduced work engagement (Maricuţoiu et al., 2017). Finally, in the current study, ED nurses scored very high on work engagement at the start of the study (T1). Although work engagement is generally seen as positive indicator of well-being, some scholars suggest a “too much of a good thing” effect (Pierce and Aguinis, 2013; Leiter, 2019). For example, high levels of work engagement in settings with high job demands can lead to over-commitment which in turn strengthens the energy-depletion process (Leiter, 2019). In line with this, high levels of work engagement are related to increased worktime demands and work-family conflict (Halbesleben et al., 2009). Still, more research is necessary to fully understand if and at what levels work engagement might be considered a negative rather than a positive aspect of employee well-being and reductions might even be considered beneficial.

Second, also against what would be expected based on the JD-R model, we found favorable changes in job demands and resources, whilst symptoms of burnout remained stable. This could be the result of the large focus on prevention during the project. Considering the high prevalence of stress-related symptoms at the beginning of the project (de Wijn et al., 2022), more focus on treating existing symptoms might be necessary to see an improvement in well-being. Furthermore, it must be noted that an absence of favorable changes on stress-related symptoms in the presence of favorable changes in job factors has been found in other stress management intervention studies conducted in the hospital setting (Le Blanc et al., 2007; Uchiyama et al., 2013; Schneider et al., 2019). These studies have two things in common. First, the programs evaluated mainly focused on improving job factors and less (or not at all) on relieving existing stress-related complaints. Second, similar to the current study, the effect on well-being in these studies is measured on rather stable outcome variables, including burnout. Although the current project encompasses a relatively long timeframe of 2.5 years, most job factors did not improve until the last year of the project. It is therefore possible that any effects of the actions taken during the project on well-being are not yet visible. Nevertheless, the current project may have been effective in preventing further deterioration of burnout symptoms. For example, in an intervention project amongst oncology care providers (Le Blanc et al., 2007), burnout levels remained stable in the intervention group but increased in the control group. Indeed, data published by the Central Bureau of Statistics shows that in general the levels of burnout amongst healthcare employees in the Netherlands increased between 2017 and 2019 (TNO and CBS, 2019). The unchanged levels of burnout in the current study thus suggest a protective effect of the actions taken by the EDs.

### Factors Related to Greater Intervention Effectiveness

Against our expectations, a multilevel approach did not lead to more favorable changes in well-being compared to an exclusively organization-directed approach. This might be explained by the person-directed part often being limited (e.g., psychoeducation on recognizing stress-related complaints and how to reduce these, a consult with the occupational health officer of the hospital) and mainly focused on prevention (e.g., implementing peer support, reducing presenteeism by stimulating employees to call in sick when experiencing stress-related complaints). In fact, out of the ten EDs using a multilevel approach, only four provided professional help for their employees (two EDs offered a mental screening followed by sessions with a trained psychologist and two offered individual coaching). Furthermore, in most EDs employees had to request additional support in order to participate in the person-directed part of the intervention. This might have increased the threshold, especially considering the still existing stigma on mental health problems within the healthcare setting (Knaak et al., 2017), resulting in a limited use of these interventions (12% of the sample between T1 and T2 and 9% between T2 and T3 reported having taken part in a person-directed intervention during the project).

Second, against our expectations, EDs that were more active (in terms of actions taken) did not show greater improvements in job factors and well-being compared to those who were less active during the project. Although activity moderated changes in staffing levels and emotional exhaustion over time, when considering the whole timeframe of the project (T1–T3) no differences were found between EDs with less of more activity. Instead, factors indicative of a more favorable implementation process including fit, communication, and employee participation in the design and implementation of actions taken were related to more favorable changes during the project. EDs with better fit of the actions to the psychosocial risks showed a greater increase in staffing levels. EDs with better communication showed greater increases in autonomy and EDs with more employee involvement showed greater increases in staffing and no increase in emotional exhaustion (a key indicator of burnout). These results are in line with previous studies stating that how interventions are designed and implemented plays a key role in the overall effectiveness of stress management interventions (Nielsen and Randall, 2013; Nielsen and Miraglia, 2016; Gray et al., 2019).

In addition, it must be noted that some moderators also related to short term negative or positive changes but that these effects were nullified when considering the entire time frame of the project. There are a number of potential explanations for these short term changes, including raised expectations and thus increased well-being which may have faded over time when changes turned out to be more limited than what was expected; a strong focus on implementing actions for short term relieve rather than designing and implementing long term solutions; or the involvement of employees in the project which often first leads to increased burden before positive effects are seen. These findings are in line with the idea that organizational change is not a linear process (Falconer, 2002) and as such confirms the current approach of using a longer timeframe to assess the effects of the project and potential moderators.

Interestingly, although communication on the intervention project was related to more job autonomy, no such effect was found for employee participation. The latter is often expected as having a say in the intervention project should automatically increase employees perceived ability to shape their own working environment. Still, mixed findings in the literature suggest that the link between employee involvement and job autonomy is more complicated than often assumed (Olsen et al., 2020). For example, a recent qualitative study suggests that if employees are involved but still perceive a limited action radius, participation will unlikely lead to the experience of more job control (Olsen et al., 2020). Since we measured participation in terms of how much employees were involved, but not the quality of this involvement (did employees felt that their ideas were heard and integrated in the actions taken), this might explain the absence of a relationship between participation and job autonomy in the current study.

Finally, half-way through the project, half of the EDs in the study participated in an intervention to create a more favorable organizational context in terms of the Psychosocial Safety Climate (PSC). It was expected that a more positive context would remove barriers and support management in the creation of more manageable job demands and adequate resources. In addition, it was expected that a more positive context would activate mechanisms related to better implementation and uptake of actions taken and as such facilitate a more effective intervention project. The results are promising, as the intervention successfully increased PSC. However, no moderating effect of (non)involvement in the PSC intervention was found on changes in job demands, resources or employee well-being over time. The late implementation of the intervention in the project resulted in a small follow-up period, which makes it difficult to draw firm conclusions regarding the influence of PSC on intervention projects. Overall, we did confirm previous research (Bronkhorst et al., 2018) that a more positive organizational context for intervention implementation can be created by means of an intervention, but a longer follow-up period is warranted to fully grasp its effects upon job factors and well-being in this setting.

### Strengths

The current study has a number of strengths. First of all, it concerns a field study including freedom for organizations to choose the number and type of actions, and how these were implemented. This made it possible to study different approaches of stress management and gives a realistic view on what can be achieved in terms of improvements in job demands, resources and well-being, within the day-to-day business of the ED. Second, the study includes a longitudinal design with an adequate timeframe to implement and study the effects of actions to reduce stress and increase employee well-being and therefore provides a good understanding of the effectiveness of stress management over time. Third, it uses a realist approach and as such leads to further understanding on how favorable results can be achieved in stress management projects. Furthermore, apart from process variables, it explored the effect of an intervention to improve the organizational context in terms of Psychosocial Safety Climate. The results are promising and might inspire future research in considering the role of contextual factors (such as PSC) in intervention projects.

### Limitations

Due to the lack of a control group, we cannot be certain that any changes in job factors and well-being were due to participation in the project and do not reflect general changes in this specific work setting. For the current project it was not feasible to establish a suitable control group as it would be incorrect to refrain EDs from taking any actions to reduce psychosocial risks for 2.5 years. Furthermore, as mentioned in the introduction, the use of randomized controlled trials to assess the effectiveness of organization-directed and multilevel interventions has received a lot of criticism (Nielsen and Noblet, 2018). As recommended (Nielsen and Noblet, 2018), we used a realist approach and focused on success factors in the project including the level of intervening and the implementation process. Finally, it must be noted that the effectiveness of the PSC intervention was assessed by comparison to a self-selected control group of EDs not partaking in this intervention.

A second limitation concerns the measurements of activity, fit and the approach (solely organization-directed or multilevel) which were depended on correct reporting of project leaders. Although follow-up telephone interviews were conducted to improve the validity of this reporting, it is possible that not all actions were listed. For example, previous research indicates that employees often report more changes compared to their line managers, suggesting that employees might also initiate own activities of which management is not aware (Hasson et al., 2012; Nielsen and Randall, 2013). In addition, since we did not have information on existing individual support programs we were not able to control for these or for support employees might have sought outside the hospital (e.g., via a general practitioner) to alleviate existing stress-related complaints. This could have influenced our findings regarding the effectiveness of a multilevel approach. Future studies might benefit from including employees viewpoints and more structured approaches to gain a more valid report of activity within an intervention project. Third, we realize that the use of a median split results in crude indicators of the moderators examined, i.e., low or high activity, fit, communication, and employee participation. Furthermore, using median-splits could have led to reduced power and therefore more conservative results in the moderation analyses (Iacobucci et al., 2015). Still, if and under what circumstances the use of a median-split increases Type I error or Type II error, or lead to reduced power, is subject of debate (DeCoster et al., 2011; Iacobucci et al., 2015; McClelland et al., 2015). Fourth, autonomy had moderate internal consistency. This is in contrast to other studies using this scale in similar populations (Adriaenssens et al., 2011, 2015). Although, the average inter-item correlation was acceptable, it is recommended to optimize this scale by including more items and differ between having autonomy on a task level or on an organizational level. Moderate internal consistency was also found for within worktime recovery. Potentially this is the result of the scale measuring short (un)official breaks as well as experiences (detachment when leaving the workplace for a short while). Future research is necessary to optimize this scale. Furthermore, as EDs implemented many actions over the course of the project, it was not possible to assess the effect for each of these actions independently. Some actions may directly influence employee well-being (person-directed), whereas others may have an indirect effect on well-being by improving job factors (organization-directed). For the latter, future research is necessary testing mediation models to understand if actions aimed at improving job factors indeed have an indirect effect on well-being as suggested by the JD-R model. Finally, the study was performed in Emergency Departments, future studies are necessary in other contexts to determine the generalizability of the current findings.

### Practical Implications

First of all, the psychosocial risk management approach (PRIMA) led to successful improvement of job demands and resources. Nevertheless, as shown in the current study, the tool reaches the greatest effects when implemented in the right way and under the right circumstances. For example, the current project emphasizes the importance of the process by which actions are designed and implemented as opposed to the number of actions taken in successfully improving working conditions and well-being. This calls for special attention for the development of fitting actions, and adequate communication and employee involvement in the intervention project. The latter can be stimulated by including employees in identifying current psychosocial risk factors in the workplace, developing actions to reduce these and evaluate the success of solutions (Glazer and Liu, 2017). Previous research indicates that employee participation in the intervention project can also be achieved by the use of employee representatives (Abildgaard et al., 2018), which seems especially advisable in a setting with high workload and high prevalence of stress related symptoms in order to avoid overburdening employees.

Second, the difficulties experienced by the EDs, including limited support from top management and limited resources (time and budget) to take action, suggests the importance of ensuring a favorable context before conducting an intervention project. PSC may be an important prerequisite, as it includes the prioritization and commitment of management to employee well-being over other competitive goals. However, more research is necessary regarding the role of PSC in intervention projects, to provide further practical recommendations.

Third although no beneficial effect of a multilevel approach over a solely organization-directed approach was found in the current study, it remains unlikely that prevention alone can alleviate existing stress-related outcomes in employees. Especially considering that stress-related outcomes such as burnout remain rather stable over time, suggesting that a self-healing process is rare (Leiter and Maslach, 2014). In settings with high prevalence of stress related outcomes, such as the ED, prevention as well as additional professional help for those with severe stress symptoms remains warranted.

Finally, most of the favorable changes in job factors but also the moderating effects of process variables occurred in the latter half of the project. This stresses the need to take into account a large timeframe when evaluating the effectiveness of this kind of intervention projects. It takes time to develop and implement actions, and effects on work factors and employee well-being may not be seen until years after the start of the project. In line with this, and as stressed by Leka et al. (2010), psychosocial risk management is not a one off activity but instead should be an ongoing cycle and includes a long term perspective.

## Conclusion

The evaluation of the current intervention project based on PRIMA (including cycles of risk assessment, designing and implementing changes, evaluating changes and adapting the approach) and participative action research in which the organizations were empowered to design and implement their own actions, shows an improvement in most job demands and job resources. Still, inclusion of person-directed interventions in the form of professional help to reduce existing stress-related complaints seem necessary to also enhance employee well-being. Furthermore, the results showed that the quality of the intervention project in terms of taking fitting actions to the psychosocial risk factors at hand, communication on the (process) of the project and employee participation in the design and development of actions, is of greater importance than the number of actions taken. This calls for more attention to the process by which actions are designed and implemented. Finally, promising results were found for an intervention to stimulate a more favorable context in terms of the Psychosocial Safety Climate. Future research may focus on the effect of higher quality multilevel interventions (including professional support for those with existing stress related complaints) and a longer follow-up period to understand how stress management interventions can effectively increase well-being.

## Data Availability Statement

The raw data supporting the conclusion of this article will be made available by the authors, without undue reservation.

## Ethics Statement

The studies involving human participants were reviewed and approved by the Psychology Research Ethics Committee from Leiden University. The patients/participants provided their written informed consent to participate in this study.

## Author Contributions

AW and MD contributed to the conception and design, acquisition of data, analysis and interpretation of data. AW conducted the interviews with management and staff. AW wrote the manuscript under supervision of MD. Both authors contributed to the article and approved the submitted version.

## Conflict of Interest

The authors declare that the research was conducted in the absence of any commercial or financial relationships that could be construed as a potential conflict of interest.

## Publisher’s Note

All claims expressed in this article are solely those of the authors and do not necessarily represent those of their affiliated organizations, or those of the publisher, the editors and the reviewers. Any product that may be evaluated in this article, or claim that may be made by its manufacturer, is not guaranteed or endorsed by the publisher.
