# Supplementary material for: Reducing Psychosocial Risk Factors and Improving Employee Well-Being in Emergency Departments: A Realist Evaluation
Source: Front Psychol. 2022 Feb 3;12:728390. doi: 10.3389/fpsyg.2021.728390 (PMC8850266; doi:10.3389/fpsyg.2021.728390)
Supplement: Supplementary file 1 [file Data_Sheet_1.PDF]

Appendix

Table 1. Overview of the steps in the intervention project based on the `psychosocial risk management approach` (PRIMA) by Leka and Cox (2010)

Preparatory actions	Establish a project group	A project group consisting of 2 researchers, 2 project managers of `Stichting IZZ` (a member collective of healthcare workers) and 1 emergency department (ED) manager was established. The project group met every two to three months to discuss the progress of the project and prepare next steps.
	Development of an occupation specific survey	The scientific literature regarding psychosocial risk factors in the ED setting was reviewed. This information was used as input for the development of an occupation specific survey to measure relevant job demands, job resources and indicators of well-being in the project.
,	Gaining management support	At the end of 2016, the study was promoted on relevant conferences/meetings and via an advertisement in the magazine of Stichting IZZ. All EDs in the Netherlands were invited to participate in the study. Next, a meeting was organized with all interested EDs to present the project in more detail. Management support was gained by informing ED management about the importance of their commitment to the project and taking actions based on the findings of the risk assessment.
	Appointing project managers	Each of the participating EDs appointed a project manager (most often the ED manager). Project managers were responsible for inventorying actions taken during the project to reduce psychosocial risks at their department, help setting up the interviews in the department and function as the first point of contact for the project group.
Step 1	Conducting a risk assessment	Work e-mail addresses and demographic variables of employees currently employed in the participating EDs were obtained from the hospital Human Resources department. Next, the online survey developed in the preparatory phase was sent to all employees from the participating EDs to measure Psychosocial Safety Climate, job demands, job resources and indicators of well-being. The survey remained open for 4-5 weeks and regular reminders were sent out. Participation in the surveys was voluntary
		Individual semi-structured interviews by the researcher with each ED manager (*k*=15) and 5-6 employees of each ED (*k*=75-90) were held to gain further insight in the most prominent psychosocial risks. Employees were randomly chosen by the researcher based upon the shift plan on the day of the interviews. The interviews were on voluntary basis and took place during worktime.
		Each ED was provided with an advisory report based upon the results of the surveys complemented by insights gained during the interviews. The report included an overview of their most prominent psychosocial risks, how to interpret these risks and a short advice regarding the main points to focus on regarding the design and implementation of actions.
Step 2	Translating risks into action plans	To support and encourage the EDs to take action, a total of nine inspiration sessions were organized by Stichting IZZ throughout the project. The sessions were open for ED management as well as employees to attend. In advance, ED managers were asked to send in any topics that they would like to see discussed during the inspiration sessions. The sessions generally consisted of a presentation on a topic of interest by an expert (e.g. "what is burnout and how to recognize it?", "how can we get psychosocial problems in the ED on the agenda of top management?", "how can we facilitate regular breaks and stimulate employees to take them?") and a presentation by one or two EDs to share a (successful) action they implemented and any barriers they encountered and how they dealt with these (best practices). The aim of these sessions was to empower the ED`s in the designing and implementation of their own actions during the project and to create a learning network.

Appendix

Step 3	Implementing interventions	EDs in the project were free to decide on the number and content of the actions they implemented. Actions were inventoried by the researchers every three to four months by means of a form to fill out and a follow-up telephone interview with the ED project manager. The form included the start date of the action, (if relevant) the end date, a description of what was implemented, the goal of the action and any comments by the project manager. Some examples of actions taken during the intervention project included: expanding the number of ED nurse trainees and supporting staff, having medical specialists working shifts on the ED during peak hours, optimizing patient flow by dividing the department in a low care and high care unit, taking security measures (e.g. doors that can only be opened by staff), psychoeducation on burnout symptoms, coaching to improve communication within the team, changing work shifts to ensure the possibility of taking breaks, and the introduction of self-rostering.<br><br>Based upon the T2 evaluation including suboptimal PSC and barriers in obtaining support and resources from hospital management for the project, a Psychosocial Safety Climate intervention was offered by Stichting IZZ (8 out 15 EDs decided to participate). The PSC intervention consisted of three steps. In the first step opinions of employees concerning the most prominent psychosocial risk factors at work were inventoried using a short online questionnaire. As the second step the team discussed the results of this poll to open a dialogue on psychosocial risks at work. In a third step, the main points from this dialogue were discussed in a meeting between employees and top management of the hospital. All steps were repeated at least three times. See Bronkhorst et al. 2018 for a detailed description of this intervention.
Step 4	Evaluating process and outcome variables	The survey of the risk assessment was repeated amongst employees at T2 and T3, assessing Psychosocial Safety Climate, job demands, job resources and well-being, complemented by additional questions to measure the process by which actions were implemented in the organization.<br><br>In addition, individual semi-structured interviews with each ED manager (*k*=15) and 5-6 employees of each ED (*k*=75-90) were held to gain insight in how actions were implemented (process variables), any changes regarding psychosocial risks, and any barriers encountered in implementing actions.<br><br>Each ED received a report including any changes in job demands, job resources and well-being in their department and the current process by which they implemented actions during the project (i.e., their scores on communication and participation). The survey findings were integrated with findings from the interviews to provide a short advice regarding the main points to focus upon in the next phase. In addition, halfway and at the end of the project the main findings were presented on one of the inspiration sessions, including an overall reflection of how the project was proceeding and a general advice on how to continue. At T2 it was strongly advised to focus more on communication on the goals and processes of the project towards employees and to involve them more in designing and implementing actions during the project. In addition, it was advised to also implement person-directed interventions to support employees with severe stress-related complaints.
